# Tolerance Mechanisms of the Aromatic and Medicinal Plant *Salvia sclarea* L. to Excess Zinc

**DOI:** 10.3390/plants10020194

**Published:** 2021-01-21

**Authors:** Anelia Dobrikova, Emilia Apostolova, Anetta Hanć, Ekaterina Yotsova, Preslava Borisova, Ilektra Sperdouli, Ioannis-Dimosthenis S. Adamakis, Michael Moustakas

**Affiliations:** 1 Institute of Biophysics and Biomedical Engineering, Bulgarian Academy of Sciences, 1113 Sofia, Bulgaria; emya@bio21.bas.bg (E.A.); ekaterina_yotsova@abv.bg (E.Y.); preslavab12345@gmail.com (P.B.); 2 Department of Trace Analysis, Faculty of Chemistry, Adam Mickiewicz University, 61-614 Poznan, Poland; anettak@amu.edu.pl; 3 Institute of Plant Breeding and Genetic Resources, Hellenic Agricultural Organisation–Demeter, Thermi, 57001 Thessaloniki, Greece; ilektras@bio.auth.gr; 4 Department of Botany, Faculty of Biology, National and Kapodistrian University of Athens, 15784 Athens, Greece; iadamaki@biol.uoa.gr; 5 Department of Botany, Aristotle University of Thessaloniki, 54124 Thessaloniki, Greece; moustak@bio.auth.gr

**Keywords:** chlorophyll fluorescence, clary sage, nutrient uptake, oxidative stress, photosynthesis, phytoremediation, phytostabilization, pigments, total phenolic content, Zn toxicity

## Abstract

In recent years, due to the development of industrial and agricultural production, heavy metal contamination has attracted increasing attention. Aromatic and medicinal plant *Salvia sclarea* L. (clary sage) is classified to zinc (Zn) accumulators and considered as a potential plant for the phytoremediation of heavy metal polluted soils. In this study, an adaptation of clary sage to 900 µM (excess) Zn exposure for eight days in a hydroponic culture was investigated. The tolerance mechanisms under excess Zn exposure were assessed by evaluating changes in the nutrient uptake, leaf pigment and phenolic content, photosynthetic activity and leaf structural characteristics. The uptake and the distribution of Zn, as well as some essential elements such as: Ca, Mg, Fe, Mn and Cu, were examined by inductively coupled plasma mass spectrometry. The results revealed that *Salvia sclarea* is a Zn-accumulator plant that tolerates significantly high toxic levels of Zn in the leaves by increasing the leaf contents of Fe, Ca and Mn ions to protect the photosynthetic function and to stimulate the photosystem I (PSI) and photosystem II (PSII) activities. The exposure of clary sage to excess Zn significantly increased the synthesis of total phenolics and anthocyanins in the leaves; these play an important role in Zn detoxification and protection against oxidative stress. The lipid peroxidation and electrolyte leakage in leaves, used as clear indicators for heavy metal damage, were slightly increased. All these data highlight that *Salvia sclarea* is an economically interesting plant for the phytoextraction and/or phytostabilization of Zn-contaminated soils.

## 1. Introduction

Heavy metals appear in the environment at high concentrations due to several industrial and agricultural activities and, subsequently, became toxic to all living organisms, including plants. Toxic metals lead to reduced plant growth, altered physiology and metabolism, as well as hamper the plant cell integrity, causing the generation of reactive oxygen species (ROS) [1,2]. Heavy metals also interfere with the uptake of essential nutrients and water, and as a result, crop yields decrease in heavy metal polluted soils [3]. In recent years, increased interest has been focused on some economically important plant species with a high capacity to accumulate heavy metals and an increased tolerance to their toxicity for the purposes of the phytoremediation of contaminated soils [2,4,5,6,7]. Plant metal accumulation varies within and between species, development stages, soil and metal types, time duration, etc. [3,4,8,9,10,11]. Baker et al. [4] proposed that hyperaccumulator species typically maintain high metal concentrations in their tissues without significant toxic symptoms using different mechanisms for detoxification and the sequestration of heavy metals in nontoxic forms [1,2,11,12,13,14,15]. 

Zinc as a micronutrient is one of the essential elements necessary for optimal growth, development and productivity, since Zn is a cofactor of many enzymes involved in the biosynthesis of plant growth hormones, respiration and photosynthesis [16,17,18]. However, Zn ions in high concentrations induce phytotoxicity, easily affecting the function of many enzymes and proteins, slowing plant metabolism and causing oxidative damage [18,19,20,21]. Visible Zn toxicity symptoms include reduced growth, leaf chlorosis (due to decreased chlorophyll content), necrosis, closure of stomata and disturbance of the water balance [22,23,24,25]. Photosynthesis is considered the primary physiological process affected by heavy metals directly or indirectly by ROS production damaging the photosynthetic apparatus of plants [23,26]. Heavy metals, including Zn, in higher concentrations also induce lipid peroxidation of the photosynthetic membranes, degrade photosynthetic pigments, inhibit photosystem II (PSII) activity and electron transport and decrease both the carboxylation efficiency of Rubisco and net photosynthesis [21,26,27,28]. The Zn toxicity first affects the chlorophyll content and then inhibits the photochemical activity of PSII [21]. In addition, the effects of Zn toxicity on the photosynthetic apparatus differ with the applied concentrations, the time of exposure, the plant species, etc. [11,23]. Chlorophyll fluorescence has been widely used as a quick and a sensitive indicator of heavy metal stress in plants [15,28,29,30,31,32,33,34,35]. 

In recent years, there has been a growing interest in aromatic plants (some herbs) that are considered suitable for environmentally safe phytoremediation, as these plants are mainly used for secondary products, and their leaf essential oils are free of heavy metals [6,36,37,38]. The aromatic and medicinal plant clary sage (*Salvia sclarea* L.) is native to many Mediterranean countries and is an important plant cultivated as a source of essential oils for applications in human medicine or perfumery products [38,39,40]. This plant is also proposed to be a Zn and cadmium (Cd) accumulator and a lead (Pb) hyperaccumulator with a good potential for phytoremediation [38,41,42]. Previously, it has been shown that heavy metals from industrial contaminated soils have almost no effect on the development of clary sage, and this plant shows no signs of heavy metal toxicity [38]. Zn effects on the nutrient uptake and functioning of the photosynthetic apparatus, as well as the tolerance mechanisms of *S. sclarea* to high Zn concentrations, have not yet been studied. Therefore, the aim of this study was to explore some of the mechanisms involved in Zn tolerance of clary sage, focusing on the investigation of changes in the uptake and distribution of essential nutrient elements (such as: Ca, Mg, Mn, Fe and Cu), as well as on some defense mechanisms that play an important role in the detoxification of high Zn levels, especially in leaves of *S. sclarea*, exposed to 900 μM Zn for eight days in a hydroponic solution. The Zn tolerance was assessed by measuring oxidative stress markers, changes in leaf photosynthetic pigments, the polyphenolic and anthocyanin contents and leaf structure, as well as by studying the functional activity of the photosynthetic apparatus (PSII and photosystem I (PSI) activities in vivo) using a chlorophyll fluorescence analysis and P700 photooxidation. 

The knowledge of these response mechanisms will be useful for the assessment of some tolerance strategies against Zn stress in this herbal plant and to optimize the management practices for phytoremediation.

## 2. Results

### 2.1. Zinc Accumulation and Mineral Element Uptake 

**Figure 1:**
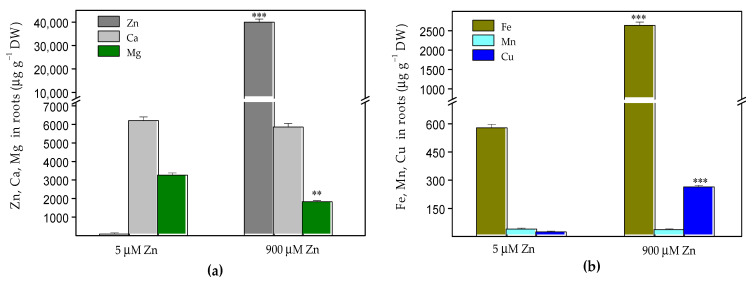
Contents of Zn, Ca and Mg (**a**) and Fe, Mn and Cu (**b**) in *Salvia sclarea* roots (µg g^−1^ dry weight (DW)) after 8 days of exposure at 5 µM (control) or 900 µM (excess) Zn. Mean values (±SE) were compared between the two Zn exposures for the same mineral element using a Student’s *t*-test, and the differences were considered statistically significant with ** *p* < 0.01 or *** *p* < 0.001.

**Figure 2:**
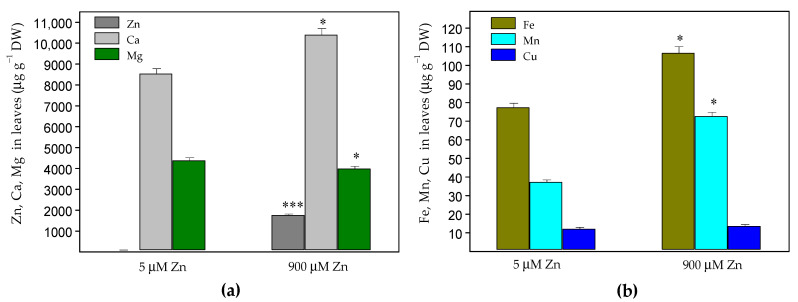
Contents of Zn, Ca and Mg (**a**) and Fe, Mn and Cu (**b**) in *Salvia sclarea* leaves (µg g^−1^ DW) after 8 days of exposure at 5 µM (control) or 900 µM (excess) Zn. Mean values (±SE) were compared between the two Zn exposures for the same mineral elements using a Student’s *t*-test, and the differences were considered statistically significant with * *p* < 0.05 or *** *p* < 0.001.

**Figure 3:**
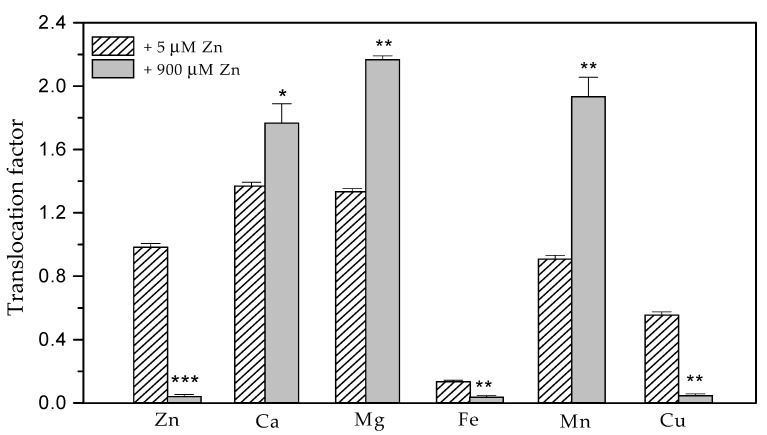
Changes in the translocation factors of the elements in *Salvia sclarea* plants in response to 5 µM (control) or 900 µM (excess) Zn exposure for 8 days. Mean values (±SE) were compared between the two Zn exposures for the same elements using a Student’s *t*-test, and the differences were considered statistically significant with * *p* < 0.05, ** *p* < 0.01 and *** *p* < 0.001.

The exposure of *Salvia sclarea* for eight days to 900 µM Zn resulted in a strongly increased Zn accumulation in both the roots and leaves. The Zn content in the roots (40,060 ± 1200 µg g^−1^ dry weight (DW), Figure 1a) was much higher than that in the leaves (1759 ± 53 µg g^−1^ DW, Figure 2a). The increased Zn uptake was accompanied with a significantly increased (about 4.4 times) accumulation of Fe and Cu in the roots (Figure 1b), as well as an increased accumulation of Fe (by 38%), Mn (by 85%) and Ca (by 22%) in the leaves compared to the control plants (Figure 2). Despite the significantly increased Cu accumulation in the roots, its translocation to the leaves decreased (Figure 3), as the leaf contents of Cu were similar to the control (Figure 2b). Under excess Zn exposure, the Mg contents in the roots decreased by 44% (Figure 1a) and in the leaves by 10% (Figure 2a), which were related with an increased translocation factor from the roots to the aboveground parts (Figure 3). In addition to the Mg ions, an increased translocation was also detected for the Ca and Mn ions (Figure 3). The translocation factor, showing a plant’s ability to translocate metal ions from its roots through the stems to the leaves [43], was less than one for the Zn, Fe and Cu ions and decreased under excess Zn (Figure 3). 

### 2.2. Oxidative Stress Markers 

Following the changes in relative water contents (RWC) and electrolyte leakages (EL) of the leaves of *S. sclarea* subjected to high Zn exposure, the impact of Zn on the leaf cell membrane integrity was evaluated. The results revealed that the Zn treatment had a slightly negative effect on the leaf membrane permeability of clary sage plants and led to increased EL values (by 22%) compared to the control leaves (Table 1) without exhibiting any other signs of toxicity (i.e., chlorosis, necrosis, rolling of leaves or disturbances in plant water-balance; Supplemental Figure S1). Additionally, oxidative stress and lipid peroxidation in clary sage leaves caused by high-level Zn exposure were estimated by the hydrogen peroxide (H_2_O_2_) and by malondialdehyde (MDA) contents as an indicator of the membrane peroxidation levels. These oxidative stress markers increased (*p* < 0.05) by about 28% for H_2_O_2_ and 21% for MDA (Table 1).

**Figure 4:**
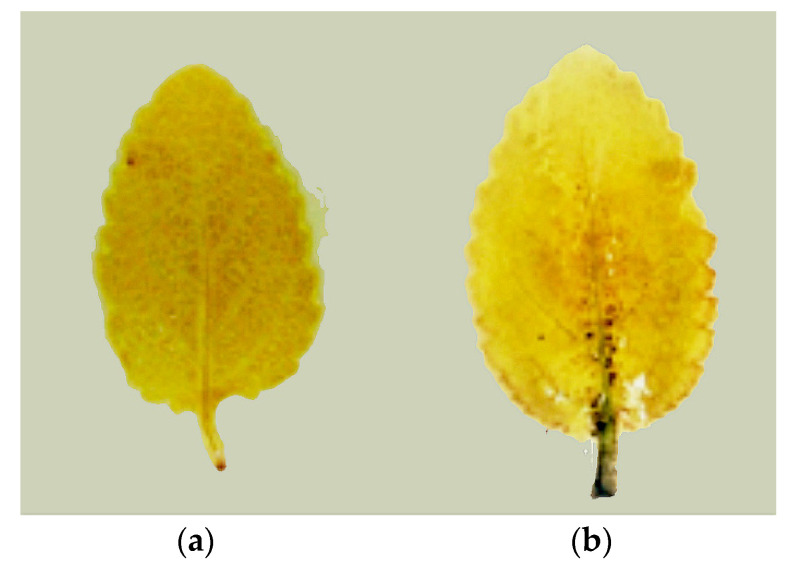
Histochemically detected hydrogen peroxide (H_2_O_2_) in the leaves of *Salvia sclarea* forming brown precipitates with 3,3′-diaminobenzidine (DAB) under (**a**) 5 µM (control) or (**b**) 900 µM (excess) Zn levels.

The histochemical detection of H_2_O_2_ overproduction in the leaves of Zn-stressed *S. sclarea* plants indicated that the high Zn exposure caused an accumulation of H_2_O_2_ mainly at the base of the leaf and around the midrib (Figure 4) without ongoing oxidative stress throughout the whole leaf. 

### 2.3. Leaf Pigments and Total Phenolic Content 

**Figure 5:**
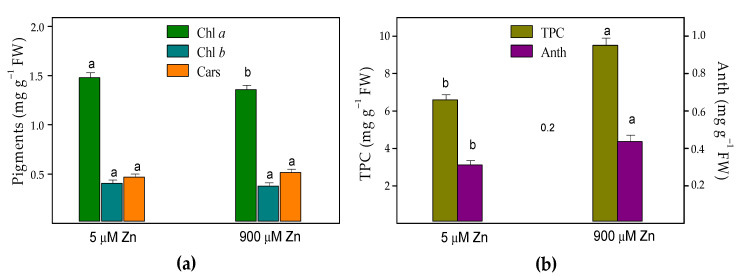
Effects of 5 µM (control) or 900 µM (excess) Zn exposure for 8 days on (**a**) the contents of the pigments: chlorophylls (Chl *a* and Chl *b*) and carotenoids (Cars) and (**b**) total phenolic contents (TPC; expressed as mg of a gallic acid equivalent per g FW) and anthocyanins (Anth; expressed as mg of a cyanidin-3-glucoside equivalent per g FW) in the leaves of *Salvia sclarea*. Different letters indicate significant differences between the mean values (±SE) for the same parameters (*p* < 0.05).

Measurements of the leaf pigments were used as sensitive biochemical markers for the metal stress and phytotoxicity. After the Zn treatment of *S. sclarea* plants for eight days, the contents of chlorophyll *a* (Chl *a*) slightly decreased by about 8%, while the contents of Chl *b* and the total carotenoids (Cars) did not change compared to those of the control leaves (Figure 5). The results also revealed that, compared to the control clary sage plants, the leaf contents of the total phenolics and anthocyanins increased under excess Zn exposure by 44% and 40%, respectively. 

### 2.4. Chlorophyll Fluorescence Analysis 

**Figure 6:**
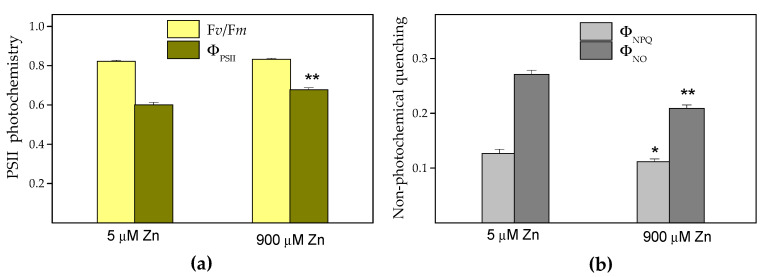
Changes in the maximum efficiency of the photosystem II (PSII) photochemistry (F*v*/F*m*) and the quantum efficiency of the PSII photochemistry (Φ_PSΙΙ_) (**a**), as well as in the quantum yield of regulated energy dissipation in PSII (Φ_NPQ_) and in the quantum yield of nonregulated energy dissipation in PSII (Φ_NO_) (**b**), of *Salvia sclarea* leaves after exposure to 5 µM (control) or 900 µM (excess) Zn for 8 days. Mean values (±SE) were compared between the two treatments for the same parameter using a Student’s *t*-test, and the differences were considered statistically significant with * *p* < 0.05 or ** *p* < 0.01.

Here, we estimated the maximum efficiency of the PSII photochemistry (F*v*/F*m*), as well as the quantum efficiency of PSII photochemistry (Φ_PSII_), the quantum yield of regulated energy dissipation in PSII (Φ_NPQ_), the quantum yield of nonregulated energy dissipation in PSII (Φ*_NO_*) and the fraction of open reaction centers (q*p*). After eight days of excessive Zn exposure (900 μM), the F*v*/F*m* did not differ compared to the control (5 μM Zn), while the Φ_PSII_ increased by about 13% (*p* < 0.01) (Figure 6a). Due to this increased Φ_PSII_, a statistically significant decrease in Φ_NO_ and Φ_NPQ_ compared to the controls was detected (Figure 6b). These three quantum yields (Φ_PSII_, Φ_NPQ_ and Φ_NO_) are equal to one, assuming that the absorbed light energy is either utilized or dissipated. The increase of Φ_PSII_ compared to the control after the eight days of Zn exposure was due to a significant (*p* < 0.01) increase in the fraction of open PSII reaction centers (q*p*) compared to the control. Under 900 μM Zn exposure, a total of 89% reaction centers were open, while under control conditions (5 µM Zn), only 79% were open.

### 2.5. P700 Photooxidation

The measurements of the steady-state P700 photooxidation (P700^+^) by far-red (FR) light-induced absorbance changes at 830 nm (Δ*A*_830_) were conducted to access changes in the PSI photochemistry of *S. sclarea* leaves after high levels of Zn exposure for eight days. The P700^+^ reduction after turning off the FR light was characterized by an exponential decay (within half the time, *t*_1/2_), as shown in our previous study [44]. The amount of P700^+^ (measured as Δ*A/A*_830_) was increased by 18% (*p* < 0.05) after the 900 µM Zn exposure in comparison to the control (Table 2). The subsequent half-time (*t*_1/2_) of the P700^+^ dark reduction was not statistically different from that in the control plants. 

### 2.6. Leaf Anatomy under Zn Stress

**Figure 7:**
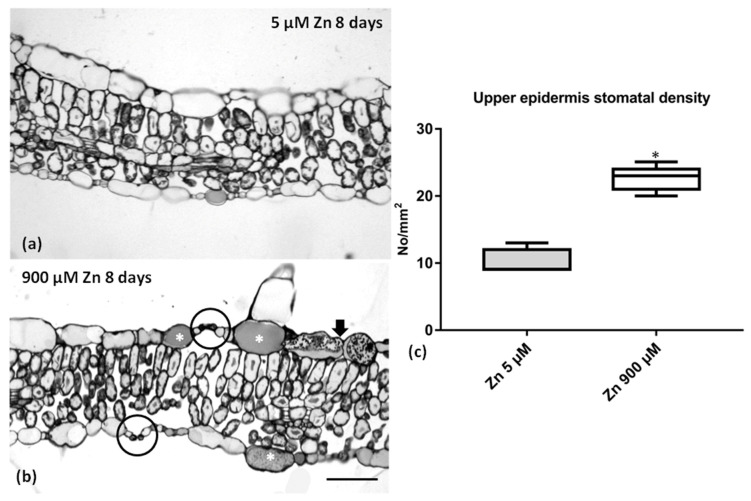
Transverse sections of *S. sclarea* leaves from (**a**) 5 μM (control) or (**b**) 900 μM (excess) Zn 8-day-treated plants. (**c**) Stomatal density (No/mm^2^) in the upper epidermis of both control and excess Zn-treated plants. Upon excess Zn application, some additional stomata could be observed (circles in (**b**)), while the epidermal cells contained darkly stained materials (asterisks and arrows in (**b**)). (**c**) Data are presented as means from sections from 4 individual leaves (* *p* < 0.05). Scale bar: 100 μm.

Excess Zn slightly affected the leaf anatomy of *S. sclarea* (Figure 7), though the leaves’ basic structures remained unaltered (compare Figure 7a with Figure 7b). However, more stomata could be observed in transverse sections (circles in Figure 7b), and the epidermal cells contained darkly stained materials (arrowheads and asterisks in Figure 7b). The stomatal density (No/mm^2^) specifically increased in the leaf upper epidermis (Figure 7c), and the stomata numbers seemed to double upon 900 μM (excess) Zn application.

## 3. Discussion 

Recently, it has been reported that *S. sclarea* grown in heavy metal-polluted areas accumulate heavy metals through the root system and then translocate them to the aboveground parts [38,42]. Our data revealed a higher accumulation of Zn in the roots than in the aboveground parts (Figure 1a and Figure 2a) and significantly decreased the translocation factor under excess Zn exposure (Figure 3). A similar decrease in the translocation of heavy metals to the aboveground parts was observed in sage plants (*Salvia officinalis*) grown in contaminated soils [45]. The decreased Zn translocation was also reported in some hyperaccumulating plants when grown hydroponically [17,34,46]. Moreover, a translocation factor of less than one suggests that plants remediate Zn by concentrating the metals in the roots [43,47]. Therefore, when grown hydroponically, *S. sclarea* phytostabilizes Zn in its roots.

The suggested Zn value for hyperaccumulation is 10,000 μg g^−1^ leaf DW (>1% DW) [4,9]. In our experimental conditions, the *S*. *sclarea* leaves did not reach these values; therefore, the clary sage can be characterized as a Zn accumulator, confirming previous observations with these plants grown in contaminated soils [38]. The presence of 8.0–100 µg Zn g^−1^ DW has been suggested to assist in the normal growth and development of plants, but higher contents above 300 µg g^−1^ DW (>0.03% DW) were considered toxic for plants [18,48] and to cause the overproduction of ROS [21]. In this study, after excess Zn exposure, we detected high Zn concentrations of about 1759 μg Zn g^−^^1^ DW (>0.17% DW) in the leaves (Figure 2a), without any symptoms of toxicity (i.e., chlorosis, necrosis, rolling of the leaves or disturbances in the RWC) (Supplemental Figure S1 and Table 1) or affecting the leaves’ structure (Figure 7). Similar high Zn concentrations were also measured in the leaves of the hyperaccumulator *Noccaea caerulescens* grown hydroponically with an 800 μM Zn supply [34]. 

Moreover, Zn can interfere with the uptake of some other trace elements, leading to an imbalance in the nutrient uptake, transport and use (see [25,34,49]). A previous study with hyperaccumulator *Noccaea caerulescens* under high Zn exposure showed a reduced uptake of Mn, Cu, Ca and Mg ions, as well as an enhanced uptake of Fe and Zn, while the Ca and Mg concentrations in the aboveground tissues remain unchanged, and the Cu increased significantly [34].

Our results demonstrated that an increased Zn uptake is accompanied with a significantly increased accumulation of Fe and Cu ions in the roots (Figure 1b), as well as increased accumulation of Fe, Mn and Ca ions in the leaves compared to the control plants (Figure 2). On the other hand, despite significantly increased Fe accumulation in roots and leaves (Figure 1b and Figure 2b), its translocation factor to the aboveground tissues is decreased (Figure 3). 

The observed increase of the nutrient elements in the leaves of *S. sclarea* (for Fe, Ca and Mn) or the maintenance of almost the same (slightly diminished for Mg) concentrations as in the control plants is most likely included in clary sage’s protective strategy against Zn stress. Moreover, the Fe, Ca and Mg cations have major roles in regulating (directly or indirectly) the photosynthetic efficiency [49]. In contrast to previous reports [50], which found no effect or an antagonistic effect of the Zn status on the Fe uptake, here, we observed a synergistic effect in the Fe uptake that suggested a strategy of an increase in Fe accumulation as a response to a possible risk of Fe deficiency in leaves (reviewed in [25]). Iron is an essential trace element required for respiration and photosynthesis and many fundamental biological redox reactions [25,51]. In the light reactions of photosynthesis, it has been also found that Fe protects the PSII from the photoinhibition that occurs under Fe deficiency [51], as well as the Fe supplement, maintaining photosynthetic electron transport [52]. Therefore, the observed increased Fe accumulation in the leaves upon 900 μM Zn exposure could be one of the reasons for the increased quantum efficiency of the PSII photochemistry (Φ_PSΙΙ_) (Figure 6a), as well as the increased photooxidation of PSI (Table 2).

Excess Zn had a significant effect on the Mg ion uptake, as the Mg contents in the roots decreased significantly (Figure 1a); however, its translocation to the aboveground parts strongly increased, leading to slightly diminished Mg leaf contents by 10% (Figure 2a and Figure 3). This was also accompanied by a slightly decreased (by 8%) amount of Chl *a* in leaves, while no noticeable changes were detected in the Chl *b* and Car contents in the leaves (Figure 5a). Recently, a significant negative correlation was reported between the Zn concentrations in the leaves and the amount of Chl *a* in *Trapa natans* L., confirming the leaf Chl *a* content as a sensitive biomarker for stress [53]. All of the above suggests a higher Zn tolerance for *S. sclarea*. At the same time, the Ca and Mn ion contents in the leaves and their translocation factors in clary sage plants were enhanced under exposure to 900 µM (excess) Zn in comparison to the control levels of Zn (5 µM) (Figure 2 and Figure 3). It has been proposed that Ca cations are necessary not only for the normal function of the oxygen-evolving complex but, also, for the regulation of Calvin cycle enzymes [54]. 

It is generally considered that ROS overproduction under heavy metal stress is a key response that can promote the lipid peroxidation of membranes, causing a disruption of their integrity (i.e., the MDA and EL increased). Therefore, the contents of H_2_O_2_ and MDA are frequently used as indicators of oxidative stress. This study provided evidence that an excess Zn treatment did not induce oxidative stress, since *S. sclarea* leaves displayed attenuated or no symptoms of toxicity, coupled with lower H_2_O_2_ and MDA contents (Table 1), and less accumulation of H_2_O_2_ in the whole leaves (Figure 4). In comparison to the control leaves, after excess Zn exposure, the H_2_O_2_ contents were higher by 28%, leading to a slightly enhanced (by 21%) lipid peroxidation (estimated as changes in the MDA contents) and EL values (Table 1), while no disturbances in the water balance (RWC) of the leaves were detected, suggesting a higher tolerance of *S. sclarea* to Zn exposure. Jin et al. [55] reported that elevated levels of Zn cause a significantly higher accumulation of H_2_O_2_ in the leaves of the non-hyperaccumulating ecotype of *Sedum alfredii*, leading to a strong increase (over five times) in the MDA contents, while, in the hyperaccumulating ecotype, this increase was less pronounced up to 1000 µM Zn. A previous study with sage plants grown in heavy metal-polluted soil suggested that the neutralization of H_2_O_2_ is a nonenzymatic rather than an enzymatic process, as indicated by the weak activities of the most antioxidant enzymes [45]. The production of H_2_O_2_ can also act as a long-distance signaling molecule activating antioxidant defense mechanisms in plants under stress [56,57]. 

It has been suggested that increased leaf phenolic compounds in some herb plants have an important role in preventing oxidative stress, thus increasing the heavy metal tolerance [58,59]. Therefore, the observed tolerance of clary sage leaves accumulating high Zn concentrations may be due to the significantly increased amounts of total phenolics and anthocyanins (Figure 5). In the leaf epidermis, the content of dark materials (Figure 7) may indicate an increase in the phenolic content. Chen et al. [60] reported a reduced Chl *a* content accompanied by a significantly increased total phenolic content in the leaves of *Kandelia obovata* under high Zn concentrations, indicating that heavy metal tolerance is related to the metabolism of phenolic compounds. Furthermore, Vidal et al. [61] confirmed that plants that produce high amounts of phenolic compounds as a response to heavy metal stress could be good candidates for phytoremediation and/or phytostabilization. Additionally, anthocyanins have also been reported to have remarkably high antioxidant capacity, acting as ROS scavengers in vacuoles and, thus, counteracting the toxic effects of heavy metals [1,62,63]. Therefore, their enhanced accumulation in clary sage leaves upon excess Zn exposure (Figure 5) indicates that a mechanism of heavy metal tolerance [64], such as the formation of anthocyanin–chelate–metal complexes in plant tissues, is also possible [65]. 

Generally, Zn excess was found to strongly affect the leaf structure. In particular, Zn-treated poplar leaves increased in thickness with their pasalidae parenchyma to substantially increase in volume [66], while in Zn-treated *Hordeum vulgare* leaves, a decrease in cell size and intercellular spaces with an increase in metal concentrations were recorded [67]. The Zn-treated *S. sclarea* plants showed none of the above effects. Both the control and excess Zn-treated leaves of *S. sclarea* had a single-layered epidermis on the upper and lower surfaces of the leaves (Figure 7). The bifacial leaves had a two to three layers of palisade parenchyma, and the spongy parenchyma consisted of irregularly shaped cells (Figure 7) having the typical anatomical features reported by Özdemir and Şenel [68]. One other interesting feature was the increase in stomatal density (Figure 7c), a phenomenon that occurred also in peanut plants under excess Zn application [69]. The increased stomata number may enhance the carbon uptake, while, at the same time, minimize the water loss [70], and this could explain the non-RWC disturbance observed (Table 1). 

Since the photosynthetic efficiency is a sensitive bioindicator of environmental stress [33], our data demonstrated stimulated PSII and PSI activity after excess Zn exposure (Figure 6a and Table 2), while there were no changes in the dark-adapted F*_v_*/F*_m_* ratio (Figure 6a) or in the O_2_ evolution (data not shown). 

The analysis of the photooxidation of P700 to P700^+^, reflecting the relative contents of active PSI reaction centers, was used to assess the effects of high Zn accumulation in leaves on the PSI activity in vivo. The level of P700^+^ is suggested to be a direct and sensitive indicator of the electron acceptance capacity from the PSI [71]. The current results revealed that the functioning of the PSI in *S. sclarea* leaves was stimulated under excess Zn exposure (Table 2). In contrast to the observed tolerance of *S. sclarea* to high Zn exposure, our recent study [72] revealed that 100 µM Cd exposure for eight days caused higher toxic effects in *S. sclarea* plants, expressed by a stronger reduction in the chlorophyll contents, as well as by an inhibition of oxygen evolution and the activities of both photosystems.

## 4. Materials and Methods 

### 4.1. Plant Growth Conditions and Zn Treatment

Seeds of clary sage (*Salvia sclarea* L., Lamiaceae) were kindly provided by Bio Cultures Ltd. (Karlovo, Bulgaria). After initial germination, seeds were sown into pots filled with soil mixed with perlite (2:1 *v*/*v*) for about 6 weeks and then were transferred for 2 weeks into hydroponic containers (3 to 4 seedlings per container) filled with a continuously aerated nutrient solution (pH 6.0) described previously in detail [35]. The seedlings were kept in greenhouse under 220 μmol m^−2^ s^−1^ photon flux density and a 12-h light photoperiod at 25/20 °C. Uniform plants were selected and subjected to treatment with 5 μM (control) or 900 μM (excess) Zn (applied as ZnSO_4_ and considered on the basis of earlier research [34]) in the nutrient solution for 8 days. For each treatment, 3 containers with four plants were prepared, and the nutrient solutions were renewed every three days. Measurements were performed on the fully expanded upper leaves of the plants.

### 4.2. Analyses of Zn and Nutrient Element Accumulation by the Inductively Coupled Plasma Mass Spectrometry Method 

After 8 days of treatment with 5 μM (control) or 900 μM (excess) Zn, roots and leaves from treated plants were harvested, washed in deionized water and dried at 75 °C to constant biomass, then milled (ball mill Pulverisette 23, Fritsch, Germany) and, finally, sieved through a polypropylene sieve. The dried tissue samples (~0.3 g) were digested in closed quartz vessels in a 3:1 ratio of 65% nitric acid and 30% hydrogen peroxide (Suprapur, Merck, Germany). The temperature of digestion was 200 °C using a microwave-assisted digestion system Ethos One (Milestone S.r.l., Sorisole BG, Italy). Digested samples were quantitatively transferred into polypropylene tubes and diluted with demineralized water (Direct-Q 3UV, Merc, Darmstadt, Germany). Elemental analysis of Zn, Ca, Cu, Fe, Mg and Mn was carried out on an ICP-MS model Elan DRC II (PerkinElmer Sciex, Toronto, ON, Canada). Spectral interference was eliminated using the dynamic reaction cell (DRC) mode with high-purity ammonia (Linde Gas, Poznań, Poland) as the reaction gas. The non-spectral interferences were reduced using a 10 ug L^−1^ solution of Ge and Rh as the internal standard. The series of standard solutions for calibration were prepared by appropriate dilution of 10 mg L^−1^ multielement stock solution (Multi-Element Calibration Standard 3, PerkinElmer Pure, Shelton, CT, USA). Calibration curves were determined by the interpolation method. The analytical procedure was validated using the certified reference material: trace elements in spinach leaves NIST SRM 1570a (National Institute of Standards and Technology, Standard Reference Material, Gaithersburg, MD, USA). More detail information about the ICP-MS operation conditions, settings and quality assurance are given in Appendix A. 

### 4.3. Determination of the Oxidative Stress Markers

For the determination of electrolyte leakage (EL), some fully expanded leaves from different selected plants were cut into small pieces and placed in 40 mL tubes with distilled water for 24 h at 25 °C in the dark. After that, the electrical conductivity of the solutions (EC1) was measured with a conductometer (Hydromat LM302, Witten, Germany); then, the samples were boiled for 30 min and cooled to 25 °C, and their final electrical conductivity was measured again (EC2). The electrolyte leakage (EL) was estimated from the equation: EL (%) = (EC1/EC2) × 100. The relative water content (RWC) of the leaves was calculated as described previously [73].

Fresh leaf samples (0.1 g) were immediately frozen in liquid nitrogen and stored at –80 °C for the analysis of the hydrogen peroxide (H_2_O_2_) and malondialdehyde (MDA) contents. The determination of the H_2_O_2_ contents in the leaves and levels of lipid peroxidation by estimating the MDA contents were made as described by Mostofa et al. [74]. The histochemical detection of H_2_O_2_ in leaves by staining with 1% 3,3′-diaminobenzidine (DAB) solution were made following the procedure described in [75]. The mean values (±SE) were averaged from three independent treatments with 3 repetitions for each treatment.

### 4.4. Analysis of Photosynthetic Pigments and Total Phenolic Content

Finely ground frozen leaf material (0.05 g) was extracted with an ice-cold 80% (*v*/*v*) acetone. The homogenates were centrifuged at 5000× *g* for 10 min at 0–4 °C, and the supernatant was measured spectrophotometrically (Specord 210 Plus, Ed. 2010, Analytik Jena AG, Germany). The amounts of chlorophyll *a* (Chl *a*), chlorophyll *b* (Chl *b*) and carotenoids (Cars) were calculated according to Lichtenthaler [76].

For an estimation of the anthocyanin and total phenolic contents, the frozen leaf samples (0.1 g) were extracted with 10 mL acidified methanol (1% HCl) in darkness at 0–4 °C for 2 days. The extracts were clarified by filtration and then used for analyses. Total phenolic content was determined by the Folin-Ciocalteu’s colorimetric method, as described by Sripakdee et al. [77]. The optical density was measured spectrophotometrically at 765 nm, and the phenolic content was expressed as mg of gallic acid equivalent (GAE) per g fresh weight (FW) of leaf tissues. Anthocyanins were estimated spectrophotometrically as the optical density of the supernatant measured at 535 and 657 nm was calculated as described by Mancinelli et al. [78]. Anthocyanin content was expressed as mg of cyanidin-3-glucoside equivalent per g FW. The mean values (±SE) were averaged from three independent treatments with 3 repetitions for each treatment.

### 4.5. Chlorophyll Fluorescence Analysis

Chlorophyll fluorescence analysis was performed using an Imaging PAM M-Series system (Heinz Walz GmbH, Effeltrich, Germany), as described in detail [79]. Measurements were conducted in dark-adapted (20 min) *Salvia sclarea* plants grown with 5 µM (control) or 900 µM (excess) Zn for 8 days. The chlorophyll fluorescence parameters that were calculated by the Imaging Win V2.41a software (Heinz Walz GmbH, Effeltrich, Germany) involved the maximum efficiency of PSII photochemistry (F*v*/F*m*), the effective quantum yield (Φ*_PSII_*), the fraction of open reaction centers (q*p*), the quantum yield of regulated nonphotochemical energy loss (Φ*_NPQ_*) and the quantum yield of nonregulated energy (Φ*_NO_*). The light intensity of 220 μmol photons m^−2^ s^−1^ was used for the photosynthetic efficiency measurements, similar to the growth light intensity.

### 4.6. Measurements of P700 Photooxidation 

The P700 photooxidation, i.e., the oxidation of the PSI reaction centers (P700) to P700^+^ [80], was measured in vivo by the far-red (FR) light-induced absorbance transients at 830 nm (Δ*A*_830_) using a PAM-101/103 fluorometer (Walz, Effeltrich, Germany) equipped with an ED-800T emitter-detector. The measurements were performed on dark-adapted leaves using FR light supplied by a photodiode (102-FR, Walz). The extent of the FR-induced oxidation of P700 to P700^+^ was estimated as the relative ratio Δ*A/A*_830_, where Δ*A*_830_ was the FR-induced absorbance change (P700^+^) and *A* was the absorbance in darkness. The capacity of the cyclic electron flow was estimated by the half-time of the P700^+^ dark reduction (*t*_1/2_) signal after switching off the FR light, as shown previously [44].

### 4.7. Light Microscopy and Stomatal Density Mesuarments

Pieces of *S. sclarea* leaves from plants exposed to 5 μM (control) or 900 μM (excess) Zn for 8 days were prepared for chemical fixation, as reported in [81]. Pieces were fixed firstly in a 3% paraformaldehyde + 3% glutaraldehyde solution buffered with 0.05 M sodium cacodylate at pH 7.0 at room temperature for 6 h, and subsequently, the leaf segments were post-fixed in 2% osmium tetroxide similarly buffered for 3 h. Afterwards, they were dehydrated in an acetone series, treated with propylene oxide and, finally, embedded in Durcupan ACM resin. An ultramicrotome (LKB 8801A, Stockholm, Sweden) equipped with a glass knife was used to obtained semi-thin sections (0.5–2 μm) that were stained with 0.5% (*w*/*v*) toluidine blue O and observed with a Zeiss Axioplan light microscope equipped with a digital AxioCam MRc 5 camera (Zeiss, Berlin, Germany). The stomatal density (No/mm^2^) was also evaluated in the leaf upper epidermis paradermal semithin sections [82] in both 5 μM and 900 μΜ Zn-exposed plants. Paradermal sections were obtained from 4 individual leaves from the central part of the leaf blade. 

### 4.8. Statistical Analysis 

Average data are presented as the mean values (±SE) of three independent experiments with three repetitions each. Statistical analysis of means was performed using two-sample Student’s *t*-tests. Differences were considered statistically significant at * *p* < 0.05, ** *p* < 0.01 and *** *p* < 0.001 by using Origin 9.0 software (OriginLab, Northampton, MA, USA).

## 5. Conclusions

To the best of our knowledge, this study revealed for the first time some of the tolerance mechanisms of the aromatic and medicinal plant *S. sclarea* to high Zn levels in the leaves, which included: (1) an altered uptake and distribution of some essential nutrients, resulting in increased contents of Fe, Ca and Mn ions in the leaves and (2) an enhanced leaf content of nonenzymatic antioxidants, such as total phenolics and anthocyanins. Our results also suggested that these mechanisms are involved into Zn detoxification and protection against oxidative damage, thus protecting the photosynthetic activity and even stimulating the PSI and PSII activities. Therefore, *S. sclarea* can be used for the environmentally safe phytoremediation/phytoextraction of Zn-contaminated soils, since this aromatic plant is mainly used for secondary products (free of heavy metals); thus, the contamination of the food chain is eliminated.

Future investigations should be focused on the details of the metabolic pathways and enzymatic antioxidant mechanisms that also contribute to the enhanced Zn tolerance in *Salvia sclarea* L. 

## Figures and Tables

**Table 1:** Effects of 5 µM (control) or 900 µM (excess) Zn exposure for 8 days on the relative water content (RWC), electrolyte leakage (EL) and the contents of hydrogen peroxide (H_2_O_2_) and malondialdehyde (MDA) in the leaves of *Salvia sclarea*.

	RWC (%)	EL (%)	H_2_O_2_ (µmol g^−1^ FW)	MDA (µmol g^−1^ FW)
5 µM Zn	93 ± 2 ^a^	9.9 ± 0.4 ^b^	34.3 ± 1.3 ^b^	25.9 ± 1.7 ^b^
900 µM Zn	89 ± 2 ^a^	12.1 ± 0.9 ^a^	43.8 ± 2.2 ^a^	31.5 ± 1.2 ^a^

Different letters indicate significant differences between the means (±SE) for the same parameters (*p* < 0.05). FW—fresh weight.

**Table 2:** Effects of 5 µM (control) or 900 µM (excess) Zn exposure on the far-red (FR) light-induced P700 photooxidation (P700^+^) and the kinetics of the P700^+^ dark reduction (half-time, *t*_1/2_) in the leaves of *Salvia sclarea*. Δ*A/A*_830_—the relative amplitudes of the P700^+^ absorbance changes at 830 nm.

	P700^+^ (Δ*A*/*A*_830_ × 10^−3^)	*t*_1/2_ (s)
5 µM Zn	11.46 ± 0.35 ^b^	2.31 ± 0.22 ^a^
900 µM Zn	13.52 ± 0.43 ^a^	1.97 ± 0.18 ^a^

Different letters indicate significant differences between the mean values (±SE) for the same parameters (*p* < 0.05).

## Data Availability

Not applicable

## Supplementary Materials

The following are available online at https://www.mdpi.com/2223-7747/10/2/194/s1: Figure S1: *Salvia sclarea* L. plants exposed to 5 μM Zn (control) or 900 μM (excess) Zn for 8 days in a hydroponic solution.

## Appendix A

### Appendix A.1. ICP-MS Operation Condition and Setting

The study was carried out using an ICP-MS spectrometer equipped with a cyclonic spray chamber, concentric nebulizer of Meinhard type, quadrupole analyzer and Pt cones. ICP-MS operation conditions were optimized daily. Those condition were: 1250 W RF power, 16 L min^−1^ plasma gas flow rate, 0.89–0.91-L min^−1^ nebulizer gas flow rate and 1.2 L min^−1^ auxiliary gas flow rate. The daily performance of ICP-MS was evaluated by the Smart Tune Solution, Elan DRC/Plus/II (PerkinElmer, Shelton, CT, USA).

### Appendix A.2. Quality Assurance

In order to provide high-quality results, the analytical procedure was validated. The parameters evaluated for the validation process included linearity, limit of detection (LOD), precision and trueness. Calibration curves were constructed daily and were based on the blank solution with an analyte addition covering a concentration range: 0–1500 μg L^−1^ for ^24^Mg, ^44^Ca and ^57^Fe and 0–100 μg L^−1^ for ^66^Zn, ^55^Mn and ^63^Cu. The correlation coefficient R exceeded a value of 0.999 for all elements. The trueness of the analytical results was evaluated through the analysis of the certified reference materials and was expressed as a recovery in percentages (R, %). The percentages of recoveries for all elements varied from 95% to 106%, respectively. The Student’s *t*-test confirmed a good agreement of trueness with the certified values for all the determined elements. The precision values, expressed as the coefficient of variation in percentages (CV, %) for all elements, were from 1.6% to 3.7%. The limit of detection (LOD) was estimated based on the standard deviation of the 10 separate blank solutions (2% HNO_3_) and the slope of the curve (b), according to the equation: LOD = 3.3S/b. The LODs estimated for the ICP-MS method were as follows: 0.01 µg g^−1^ (Zn), 30 µg g^−1^ (Ca), 0.05 μg g^−1^ (Cu), 40 μg g^−1^ (Fe), 0.8 μg g^−1^ (Mg) and 0.03 μg g^−1^ (Mn) [72].
