# Supplementary material for: Tolerance Mechanisms of the Aromatic and Medicinal Plant Salvia sclarea L. to Excess Zinc
Source: Plants (Basel). 2021 Jan 21;10(2):194. doi: 10.3390/plants10020194 (PMC7909794; doi:10.3390/plants10020194)
Supplement: Supplementary file 1 [file plants-10-00194-s001.pdf]

Tolerance Mechanisms of the Aromatic and Medicinal Plant *Salvia sclarea* L. to Excess Zinc

Anelia Dobrikova, Emilia Apostolova, Anetta Hanć, Ekaterina Yotsova, Preslava Borisova, Ilektra Sperdouli, Ioannis-Dimosthenis S. Adamakis and Michael Moustakas

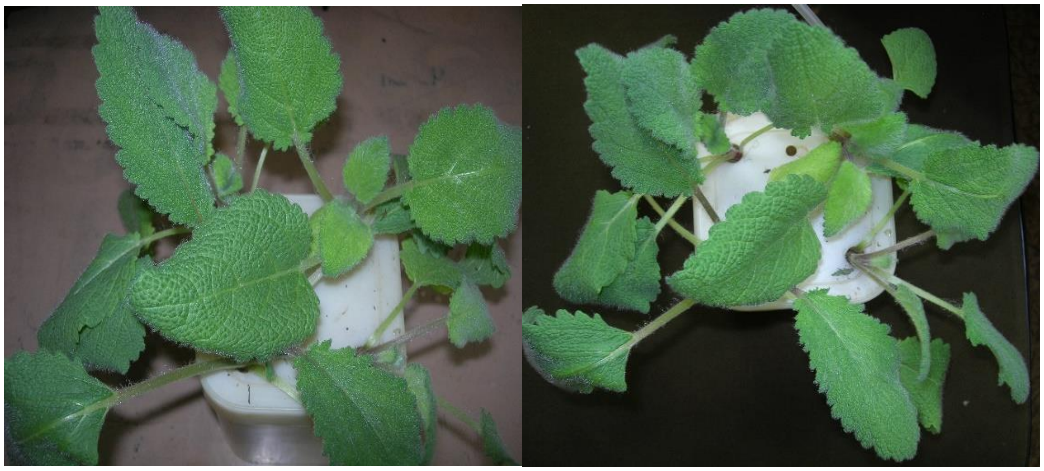

5 µM Zn 900 µM Zn

Figure S1. *Salvia sclarea* plants exposed to 5 µM (control) or 900 µM (excess) Zn for 8 days in a hydroponic solution.
